# An Update on Refractory Hypertension

**DOI:** 10.1007/s11906-022-01185-6

**Published:** 2022-04-06

**Authors:** Faris Matanes, M. Bilal Khan, Mohammed Siddiqui, Tanja Dudenbostel, David Calhoun, Suzanne Oparil

**Affiliations:** Vascular Biology and Hypertension Program, University of Alabama at Birmingham, Birmingham, AL 35294-2180 USA

**Keywords:** Sympathetic activation, Primary aldosteronism, Sleep apnea, Spironolactone, Renal denervation

## Abstract

**Purpose of Review:**

To update on definition, diagnosis, prevalence, patient characteristics, pathophysiology, and treatment of refractory hypertension (RfHTN).

**Recent Findings:**

Refractory hypertension (RfHTN) is defined as blood pressure (BP) that is uncontrolled despite using ≥ 5 antihypertensive medications of different classes, including a long-acting thiazide diuretic and a mineralocorticoid receptor antagonist (MRA) at maximal or maximally tolerated doses. This new phenotype is different from resistant hypertension (RHTN), defined as BP that is uncontrolled despite using ≥ 3 medications, commonly a long-acting calcium channel blocker (CCB), a blocker of the renin-angiotensin system (angiotensin-converting enzyme [ACE] inhibitor or angiotensin receptor blocker [ARB]), and a diuretic. The RHTN phenotype includes controlled RHTN, BP that is controlled on 4 or more medications. RfHTN is largely attributable to increased sympathetic activity, unlike RHTN, which is mainly due to increased intravascular fluid volume frequently caused by hyperaldosteronism and chronic excessive sodium ingestion. Compared to those with controlled RHTN, patients with RfHTN have a higher prevalence of target organ damage and do not have elevated aldosterone levels. Ongoing clinical trials are assessing the safety and efficacy of using devices to aid with BP control in patients with RfHTN.

**Summary:**

RfHTN is a separate entity from RHTN and is generally attributable to increased sympathetic activity.

## Introduction

**Fig. 1:**
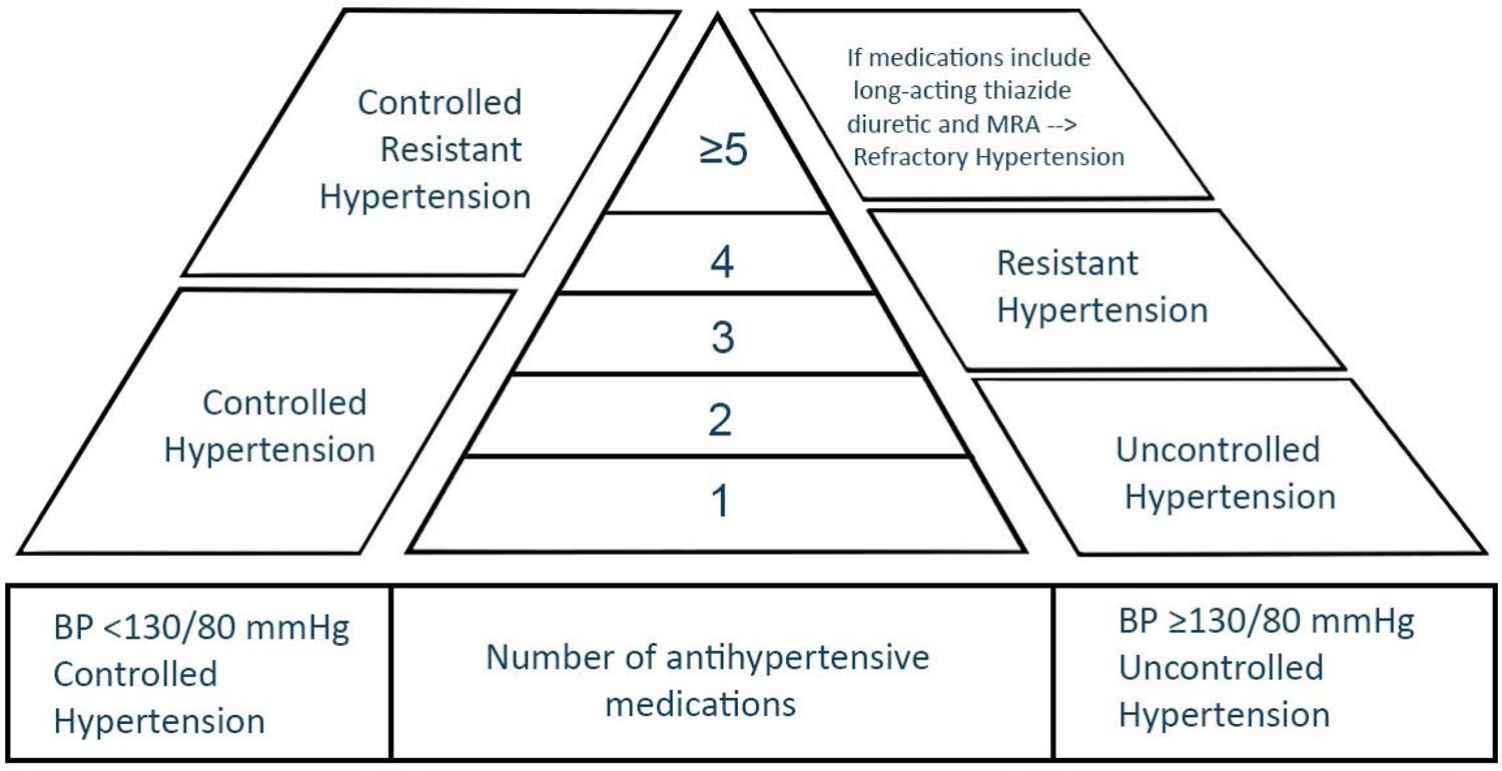
Graph illustrating the phenotypes of hypertension

Resistant hypertension (RHTN) is defined as BP that is uncontrolled despite using ≥ 3 medications of different classes, commonly a long-acting calcium channel blocker (CCB), a blocker of the renin-angiotensin system (angiotensin-converting enzyme [ACE] inhibitor or angiotensin receptor blocker [ARB]) and a diuretic. The definition also includes controlled RHTN, BP that is controlled on 4 or more medications (Fig. 1). All agents should be administered at maximum or maximally tolerated doses and at the appropriate dosing frequency [1••]. RHTN is generally attributable to persistent fluid retention [2, 3], secondary, in large part, to hyperaldosteronism and chronic excessive sodium ingestion [4]. Recent studies indicate that the key to overcoming chronic fluid retention in RHTN is using combination diuretic therapy, specifically a long-acting thiazide-like diuretic in combination with a mineralocorticoid receptor antagonist (MRA) [5•, 6]. The refractory hypertension (RfHTN) phenotype refers to patients whose BP remains uncontrolled despite use of maximal or near maximal antihypertensive therapy [7••, 8••]. This phenotype represents a subgroup of patients with RHTN who are at an extremely high risk of cardiovascular disease (CVD) complications because of their history of longstanding, poorly controlled, and often severe hypertension (HTN). This review will provide a comprehensive update of the phenotype, including definition, prevalence, patient characteristics, prognosis, and insight into the underlying pathophysiology.

## Definition

RfHTN is defined as BP that is uncontrolled despite using ≥ 5 different antihypertensive agents, including a long-acting thiazide diuretic (i.e., chlorthalidone) and an MRA (spironolactone or eplerenone) [7••, 8••]. For patients to be diagnosed with RfHTN, they should be treated with maximal doses of these medications and secondary causes of HTN should have been ruled out or, if diagnosed, should have been treated appropriately. Requiring the use of a thiazide-like diuretic and an MRA is critical to establish true treatment resistance. Intravascular fluid retention and hyperaldosteronism are commonly found in patients with RHTN, and thiazide diuretics and MRAs are effective in treating these conditions. Hence, patients who have uncontrolled BP despite taking these medications are classified as having RfHTN, which is attributable to other mechanisms, including increased sympathetic activity.

### True Treatment Resistance

To classify a patient as having RHTN or RfHTN, it is important to ensure that all of the following conditions are met:

1. All antihypertensive medications are prescribed at maximal or maximally tolerated doses.
2. The patient is adherent to all medications.
3. White coat effect is ruled out.

### White Coat Effect

White coat effect is defined as BP that is uncontrolled in clinic but controlled or significantly lower out-of-clinic in a patient taking antihypertensive medications. A study from our Hypertension Clinic investigated the prevalence of white coat effect in patients with RfHTN. Patients were enrolled after having clinic BP ≥ 135/85 mmHg for 3 consecutive clinic visits. Twenty-four-hour ambulatory BP monitoring (ABPM) readings were obtained every 20 min during daytime and every 30 min during nighttime. The prevalence of white coat effect was only 6.5% (2 out of 31 patients) [9] (Table 1).

### Adherence

Difficulty in assessing adherence to medications is a major factor that hinders both phenotypic classification and management of patients with difficult to manage HTN. A recent study from our group assessed medication adherence in patients with RfHTN by measuring antihypertensive medications and their metabolites in 24 h urine specimens using high-performance liquid chromatography-tandem mass spectrometry. Of 40 patients with RfHTN, only 16 (40%) had complete adherence with all prescribed medications, 18 (45%) had partial adherence, and 6 (15%) were completely non-adherent with all prescribed medications. Overall, 21 (52.5%) were adherent with 5 or more medications, including chlorthalidone and an MRA [10••]. This is comparable to levels of adherence in other HTN phenotypes. A study in Germany assessed adherence in patients with RHTN, defined as BP ≥ 140/90 mmHg and/or 24 h ABPM ≥ 130/80 mmHg despite the use of 3 or more medications, including a diuretic, using detection of antihypertensive medications or their metabolites in urine. The results showed similar adherence to that of patients with RfHTN: 47.4% were adherent to all medications, 37% were adherent to some of the prescribed medications, and 15.8% took none of the prescribed medications [11].

## Prevalence and Patient Characteristics

A large population-based study using the National Health and Nutrition Examination Surveys (NHANES) database has shown that of 12,389 persons who were taking antihypertensive medications, only 0.8% were classified as having RfHTN. Persons with RfHTN were more likely to be elderly, African American, and had lower income [12]. RfHTN in that study was defined as elevated BP despite taking ≥ 5 antihypertensive medications, including a diuretic, and white coat effect was not ruled out. Other studies carried out in patient populations in the USA and other countries have shown that the age and gender of patients with RfHTN differ from those reported using NHANES data [8••, 13]. Patients with RfHTN were more likely to be young women in a study by Dudenbostel et al. [8••] in patients in the USA, and of black race in a study by Modolo et al. in patients in Brazil [13]. The study of Dudenbostel et al. found no difference in the prevalence of chronic kidney disease, diabetes, stroke, or CAD between patients with RfHTN and those with controlled RHTN. The differences between studies in the characteristics of RfHTN patients may be related to the nature of the comparator groups. The study of Buhnerkempe et al. was population based and used NHANES data to compare patients with RfHTN to the general population. Modolo et al. compared patients with RfHTN to those with RHTN (defined as patients whose BP is uncontrolled despite using 5 or more medications—spironolactone or chlorthalidone use was not included in the definition). Dudenbostel et al. compared patients with RfHTN to those with RHTN and did require use of spironolactone 25 mg per day and chlorthalidone 25 mg per day for the definition of RfHTN. The latter two studies were carried out in patients referred to a specialty Resistant Hypertension Clinic in a tertiary medical center.

## Pathophysiology

Recent studies have provided evidence that the pathophysiology of BP elevation may differ between patients with RfHTN and those with controlled RHTN. Dudenbostel et al. studied 15 patients with RfHTN and 29 with controlled RHTN who were enrolled after having met the definitions of RfHTN or controlled RHTN after follow-up of at least 3 clinic visits over 6 or more months. All participants had 24 h urine collection and measurement of pulse wave velocity (PWV) and impedance cardiography. Patients with RfHTN had higher 24 h urinary normetanephrine levels than those with controlled RHTN (464.4 ± 250 versus 309.8 ± 147.6 μg; *p* = 0.039) [8••]. Daytime and nighttime heart rates were higher, and heart rate variability was less in RfHTN patients. These findings suggest that increased sympathetic activity is the underlying pathophysiologic mechanism of RfHTN. Furthermore, arterial stiffness indexed by carotid-femoral PWV, the gold standard for measuring arterial stiffness, was also higher in the RfHTN group [14].

In the same study, thoracic fluid content, indexed by thoracic impedance cardiography (ICG), was similar in RfHTN patients and those with controlled RHTN, suggesting that thoracic fluid retention did not contribute to treatment resistance [8••]. ICG also demonstrated higher systemic vascular resistance in patients with RfHTN, further supporting increased sympathetic activity as an underlying pathophysiologic mechanism [8••]. Velasco et al. performed cardiac magnetic resonance (CMR) imaging to assess intravascular fluid volumes and cardiac anatomy and function in 24 patients with RfHTN and 30 with controlled RHTN. Participants were enrolled after qualifying as having RfHTN or controlled RHTN according to their office BP and number of antihypertensive medications over 3 consecutive clinic visits. They were required to be taking chlorthalidone and an MRA (spironolactone or eplerenone) to qualify as having RfHTN. Both groups had similar left atrial and ventricular volumes and similar B-type natriuretic peptide levels [15•]. Participants in the studies of Dudenbostel et al. and Velasco et al. were on maximal tolerated doses of antihypertensive medications, had white coat effect ruled out using ABPM, and were assessed for medication adherence, thus ensuring true treatment resistance. In the study by Dudenbostel et al., patients were excluded if they scored > 2 on Morisky 8-Item adherence Questionnaire. RHTN has been attributed to increased intravascular fluid volume [2, 3], but results from our recent studies provide evidence that increased intravascular fluid volume is not the main pathophysiologic mechanism underlying RfHTN [8••, 15•].

## Comorbidities and Target Organ Damage

Data from the NHANES database show that patients with RfHTN are more likely to have lower estimated glomerular filtration rate (eGFR), higher prevalence and severity of albuminuria, and higher prevalence of diabetes, prior stroke and coronary artery disease (CAD) when compared to patients with RHTN [12]. RfHTN in that study was defined as elevated BP despite taking ≥ 5 antihypertensive medications, including a diuretic. Furthermore, studies from our HTN specialty clinic using the new definition of RfHTN with true treatment resistance show that patients with RfHTN had a higher prevalence of diabetes mellitus and heart failure, as well as a higher mean body mass index, compared to patients with controlled RHTN [8••, 15•]. Other comorbidities, such as coronary artery disease, prior cerebrovascular events, and obstructive sleep apnea (confirmed by polysomnography), were similar in both groups [15•].

Cardiac remodeling is one of the most common complications of hypertension. Left ventricular hypertrophy and diastolic dysfunction, in particular, are associated with adverse CVD outcomes in both hypertensive and normotensive individuals [16, 17]. In the study by Velasco et al., patients with RfHTN had higher left ventricular (LV) mass indexed by body surface area than patients with controlled RHTN (88.3 ± 35.0 versus 54.6 ± 12.5 g/m2; *p* < 0.0001) [15•]. Interventricular septal thickness and posterior wall thickness were also significantly greater in patients with RfHTN, and these patients had higher LV mass/LV end diastolic volume ratios (1.3 ± 0.4 versus 0.8 ± 0.2 g/mL; *p* < 0.0001). Left ventricular diastolic function, assessed using peak filling rate and time needed in diastole to recover 80% of stroke volume, was also more impaired in the RfHTN group.

## Evaluation

Having RHTN or RfHTN is one of the indications for evaluation for secondary causes of HTN [18••]. The 2017 HTN guidelines recommend initially screening for secondary HTN by gathering clues from the history and physical examination. Specific laboratory testing and imaging should then be performed based on the suspected etiology. The most common causes of secondary HTN are obstructive sleep apnea (OSA), primary aldosteronism, renovascular disease, renal parenchymal disease, and alcohol or drug-induced [18••].

### Primary Aldosteronism in RfHTN

Patients with RHTN frequently have elevated aldosterone levels and a high prevalence of primary hyperaldosteronism [4, 19]. In contrast, patients with RfHTN have 24 h urinary aldosterone levels similar to those of patients with controlled RHTN (11.6 ± 8 versus 12.3 ± 7 μg/24 h; *p* = 0.829) and similar levels of plasma renin activity [8••]. Participants in this study were enrolled after having uncontrolled clinic BP after 3 or more follow-up visits over a 6-month period while taking chlorthalidone and spironolactone. White coat effect was ruled out by 24 h ABPM, and urinary aldosterone levels measured at the first study visit before spironolactone was started were elevated. Since patients with RfHTN, by definition, have uncontrolled BP despite being treated with a diuretic and spironolactone, the latter being the medical management method of choice for primary hyperaldosteronism, it is unlikely that the cause of the uncontrolled BP in these patients was aldosterone excess.

Recent evidence shows that hyperaldosteronism is not simply a consequence of having an elevated aldosterone–renin ratio, but rather is characterized by a spectrum of increased aldosterone production independent of renin [20•]. A multicenter study investigated 24 h urinary aldosterone excretion, plasma renin activity and biomarkers of mineralocorticoid receptor activation such as urine potassium to sodium ratio and serum potassium in patients with untreated normal BP, untreated stage I and II HTN and RHTN treated with a variety of antihypertensive medications. Using suppressed levels of renin in the context of high sodium intake instead of simply using a cutoff of 24 h urinary aldosterone excretion resulted in better detection of primary aldosteronism in all groups. There was a linear relationship between HTN severity and renin-independent aldosterone levels. This new definition of primary aldosteronism was characterized by the presence of biomarkers of mineralocorticoid receptor activation. This new evidence may lead to a redefinition of primary aldosteronism and shed new light on the pathophysiology of the various phenotypes on HTN.

## Management

### Medical

Choice of the four preferred classes of medications, CCB, ACE inhibitor or ARB, long-acting thiazide, or thiazide-like diuretic and MRA, for the general population of hypertensive patients is based on the reductions in CVD morbidity and mortality that have been observed in clinical trials of these agents [21–26]. Recommended medications for hypertension after those four, based on expert opinion, are the following: beta blockers, central alpha 2 agonists (e.g., clonidine and guanfacine) and vasodilators (e.g., hydralazine and minoxidil) [1••]. The choice among these medications is also based on heart rate: unless the heart rate is < 70 beats/min, beta blockers are indicated. If there is a contraindication to beta blockers, central alpha 2 agonists can be used. History of medication intolerance should also be taken into account. Clinical trial evidence of CVD outcome benefit from these medications in hypertensive patients is lacking unless other comorbidities, e.g., heart failure or arrhythmias are present [27, 28].

A recent proof of concept study from our group enrolled 6 patients with RfHTN with uncontrolled BP in clinic (measured by automated office blood pressure [AOBP] and confirmed with ABPM) and administered reserpine, a sympatholytic agent, to them [29]. Participants were offered enrollment into the study after medication adherence was confirmed by testing for drugs and drug metabolites in the urine. Sympatholytic agents such as clonidine and guanfacine were tapered off prior to enrollment. A total of 67.7% of the patients were female and all were African American. After 4 weeks of therapy with reserpine 0.1 mg per day, the patients had average reductions of 29.3 ± 22.2 mm Hg in systolic AOBP and 21.8 ± 13.4 mm Hg in ABPM systolic BP. Although this study was open label, uncontrolled and had only 6 participants, it showed that targeting increased sympathetic activity in RfHTN can result in large, sustained BP reductions. Further studies, which include additional data on the safety and adverse effects of this approach, are needed to confirm these preliminary findings.

### Device-Based Interventions

Since by definition, patients with RfHTN have failed medical treatment, device-based interventions have been developed in an attempt to reduce BP and prevent adverse CVD outcomes in these patients. Baroreflex activation therapy (BAT), renal denervation (RDN), and continuous positive airway pressure (CPAP) have been and are being used in clinical trials to control BP in patients with RHTN and RfHTN.

### Baroreflex Activation Therapy

BAT uses electrical impulses from a pulse generator to decrease sympathetic nervous system activity and promote vagal nerve activity, resulting in arterial vasodilation and reduced heart rate. Since increased sympathetic nervous system activity likely plays an important role in the pathophysiology of RfHTN, this intervention would likely be helpful in these patients. A proof-of-concept study has shown a good safety profile and reduction in BP, heart rate, left ventricular mass, and thickness all after 1 year of follow-up in patients with RHTN [30]. The first randomized clinical trial of BAT divided patients into two groups [31]. The first group received BAT for 12 months and the second had the device turned off for the first 6 months then activated for the following 6 months. Results showed an average decrease of 26 ± 30 mmHg and 17 ± 29 mmHg in SBP for the two groups, respectively, after 6 months (*p* = 0.03). The acute efficacy endpoint (proportion of participants in the treatment group with a SBP reduction ≥ 10 mmHg is 20% or greater than that in the placebo group) was not met in this study. Actual percentages were 54% and 46%, in the first and second groups, respectively. The study did not meet the pre-specified procedural safety endpoint, an event-free rate of 82%; the actual rate was 74.8%. The most common adverse effect related to the procedure was nerve damage during device implantation. The first group had a 40% decrease in incidence of hypertensive crisis compared to the second group. The percentages of patients on 5 or more antihypertensive medications in these groups were 65% and 58%, respectively. Only 17% of patients in the first group and 19% in the second group were taking an MRA [31]. A 6-year follow-up of the device showed a sustained reduction in BP (average reduction > 30 mmHg in SBP and 15 mmHg in DBP) and a good safety profile [32].

A second-generation BAT device (BAT neo) showed an improved safety profile in a pivotal trial [33]. An observational study showed that patients with RHTN who had the second-generation device inserted had a 25 ± 33 mmHg reduction in clinic SBP after 24 months, despite reduction in the median number of medications from 7 to 5 [34]. An ongoing trial in Europe is testing this device in patients with RHTN [35]. The trial in the USA was recently suspended “as company resources will only allow adequate oversight for one pivotal trial at a time” [36]. A national registry in the United Kingdom is also being established to follow up patients undergoing BAT neo [37]. It is important to note that the trials of the second-generation device are not randomized controlled trials, and results of such trials are needed to fully evaluate the potential benefits of the procedure. Advantages of BAT include the ability to quickly assess the magnitude BP reduction and the full reversibility of its effects. However, questions about safety and whether the second-generation devices are safer and more effective than the first-generation devices are yet to be addressed. In addition, analyzing the results of BAT for patients with RfHTN separately might be especially informative, as this treatment targets increased sympathetic activation.

### Continuous Positive Airway Pressure

Apneas and hypopneas that are characteristic of OSA have been reported to cause increased sympathetic activity [38], perhaps accounting for the association between OSA and RfHTN. Although OSA has been reported to be equally prevalent in some populations of RfHTN and controlled RHTN patients [8••, 15•], a large multi-center study recently found that patients with RfHTN had a higher prevalence of OSA and more severe OSA than RHTN patients [39]. Use of CPAP, the treatment of choice for patients with OSA, for 3 months has been shown to result in reductions in DBP, especially at night, in patients with both RHTN and RfHTN. The analysis also showed a significant decrease (9 mmHg) in SBP in the RfHTN group, but not in the RHTN group [40]. Neither of these studies specified the need for use of long-acting thiazide diuretics or MRAs to define RfHTN.

In contrast, a multicenter study showed that using CPAP to treat OSA in patients with CVD did not reduce CVD events. About 78% of the patients in that study had hypertension, although the numbers of antihypertensive medications were not specified and the BP values did not change in the intervention arm during CPAP treatment [41]. Although some studies confirmed a lack of reduction in CVD outcomes with CPAP treatment overall, two studies showed that the subset of patients who were more compliant with CPAP (≥ 4 h per night) had significant reductions in both new onset hypertension and CVD outcomes [42, 43]. This affirms the caveat that one of the greatest obstacles in interpretation of trials of the effects of CPAP on CVD outcomes is variable compliance with the treatment.

### Renal Denervation

Efferent renal nerve activation enhances renin secretion, leading to activation of the renin-angiotensin system and an increase in BP [44]. Afferent renal nerve activation has a central effect that increases sympathetic activity as well [45]. Renal denervation (RDN) can decrease the activity of both efferent and afferent renal nerves, but the efficacy of renal nerve denervation as a treatment for patients with RHTN and RfHTN is debated. Three large multi-center trials have shown conflicting results. The Symplicity HTN 1 and 2 studies showed reductions in BP that were sustained over 3 years of follow-up [46, 47], while Symplicity HTN-3 showed no significant reduction in BP [48]. All three studies used the Symplicity radiofrequency catheter and participants in in both the treatment and sham groups of all three trials were taking 5 antihypertensive medications on average. In the Symplicity HTN-3 trial, the change in office BP at 6 month of follow-up was − 14.13 ± 23.93 mm Hg in the denervation group compared to −11.74 ± 25.94 mm Hg in the sham-procedure group (*p* = 0.26) [49••].

Importantly, many aspects of the Symplicity HTN-3 trial may have masked a possible benefit of denervation on BP [49••]. A post hoc analysis showed that only 6% of participants had successful bilateral ablation in 4 quadrants of the arteries. Furthermore, some patients benefited more than others. African Americans who had the procedure did not have a significant BP reduction and patients of non-African American descent who were not on vasodilators at the beginning of the study had significantly greater decreases in BP. Furthermore, use of an MRA and having baseline office SBP ≥ 180 mmHg were independent predictors of greater reduction in BP after 6 months of follow-up [49••]. This suggests that patients who have RfHTN might be more responsive to RDN than patients with RHTN. Furthermore, patients who received 4 quadrant ablations on both renal arteries achieved a mean office BP reduction of 24.3 mmHg compared to 16.1 mmHg and 14.2 mmHg reductions in those who had a 4-quadrant ablation on one side or no four-quadrant ablation on either side. However, the differences among these three groups were not statistically significant [49••]. An extended follow-up of patients in the Symplicity HTN-3 trial showed that the reduction in SBP was significantly greater after 1 year compared to 6 months after the procedure (−18.9 ± 25 vs. −15.5 ± 24.1 mmHg; *p* = 0.025). In addition, a subset of patients in the control group underwent denervation and had a 17.7 ± 23.2 mm Hg reduction in SBP from baseline following the procedure (*p* < 0.001) [50].

The RADIOSOUND-HTN trial compared the BP effects of ultrasound-based ablation of the main renal artery (USM), radiofrequency ablation of the main renal artery (RFM), and radiofrequency ablation of the main renal artery, side branches and accessories (RFB). The trial showed that USM was superior to RFM (reduction in mean daytime ABPM of 13.2 ± 13.7 versus –6.5 ± 10.3 mm Hg respectively; adjusted *p* = 0.043) [51]. RFB reduced BP slightly more than RFM, but the difference was not statistically significant. Approximately half of the patients in the study were treated with 5 or more antihypertensive medications, and 23% were on an MRA.

An expert panel recently concluded that using baseline patient characteristics to predict which patients will have the greatest BP reduction to device therapy will help address the wide variation in BP reduction seen in the earlier trials and provide a better estimate of its true efficacy [52••]. The only factor that predicted reduction in response to denervation across multiple studies in this report was baseline BP [52••]. Other patient characteristics that had previously been shown to predict denervation-induced BP reductions, but were not seen consistently in all studies, include the following: younger age [48], Caucasian race [49••], number and classes of antihypertensive medications at baseline [49••], obesity [53], number of successful ablations [49••], higher baseline heart rate [54], higher mean and standard deviation of nighttime ABPM readings [55], and low pulse wave velocity [56, 57]. Importantly, the studies that identified these predictors were carried out in patients with a variety of HTN phenotypes, not only RfHTN. Furthermore, evidence of the efficacy of renal denervation in reducing CVD outcomes is limited to retrospective studies [58]. Prospective studies assessing the effects of renal denervation on organ damage are urgently needed.

Combined data from published studies show that the method of RDN, completeness of renal nerve ablation and patient characteristics all play important roles in determining the extent of the resultant decrease in BP. The finding that RDN reduced BP by < 10 mmHg in most of these published reports may be due to patient characteristics and the devices used. Future studies may show more impressive results if these issues can be addressed.

## Outcomes

A recent review paper has summarized the worse prognosis of patients with RfHTN based on 2 large prospective studies [59]. The first study enrolled patients seen over 16 years in outpatient clinics in a university hospital in Brazil [60]. The study defined RfHTN as uncontrolled BP based on clinic BP ≥ 130/80 mm Hg and 24 h ABPM ≥ 125/75 mm Hg despite using 5 or more antihypertensive medications including a diuretic and an MRA. Compared to patients without RfHTN, those with RfHTN had increased hazard ratios (HR) for major adverse cardiovascular events 1.52 (1.08–2.16); stroke 1.86 (1.05–3.28), cardiovascular mortality 1.75 (1.15–2.64), and all-cause mortality 1.46 (1.03–2.07) based on clinic BP. These HR were calculated after adjusting for age, sex, body mass index, smoking status, diabetes mellitus, history of CVD and eGFR. The other study used patients from the Chronic Renal Insufficiency Cohort in the USA and defined RfHTN as clinic BP ≥ 140/90 mmHg despite taking ≥ 5 antihypertensive medications, including a diuretic [61]. MRA was not included in the criteria due to infrequent use in the study population and ABPM was not available in the study. Patients with RfHTN had increased HR for the composite of stroke, myocardial infarction, and congestive heart failure 3.51 (1.71–7.19) and composite renal outcomes (50% reduction in eGFR or developing end-stage renal disease) 1.66 (1.31–2.10) compared to those with uncontrolled RHTN over a maximum of 10 years of follow-up. The mean follow-up time was approximately 7 years. These HRs were calculated after adjusting for sex, race, age, smoking status, eGFR, low-density lipoprotein levels and history of CVD, among other variables.

## Conclusion

This review discusses the evolution of the definition of RfHTN. It is critical to properly phenotype these patients and identify evidence-based effective treatment for them since they are at greatly increased risk of target organ damage. Ensuring true treatment resistance by assessing adherence, maximally tolerated medication dosing and ruling out white coat effect should be carried out in all patients. A major limitation in interpreting the results of clinical trials of RHTN and RfHTN treatment is the inclusion criteria for these trials, which in many cases do not strictly satisfy the new definitions of RHTN and RfHTN. We propose that future trials should assess whether patients who properly fit the definitions of these phenotypes have different treatment responses from patients who do not. Since patients with RHTN and RfHTN usually have an underlying pathophysiology that differs from that in patients with other phenotypes of HTN, they may respond differently to new medications and interventions. Applying a precision medicine approach by addressing the underlying pathophysiology that is responsible for resistance to medical and device-based BP treatment would improve BP control and outcomes in these patients.

**Table 1:** White coat and masked effects in hypertension

	BP in clinic	BP out-of-clinic
White coat effect	Above goal	At or below goal
Masked hypertension	At or below goal	Above goal
